# Synthesis of hybrid amorphous/crystalline SnO_2_ 1D nanostructures: investigation of morphology, structure and optical properties

**DOI:** 10.1038/s41598-020-71383-2

**Published:** 2020-09-09

**Authors:** Wiktor Matysiak, Tomasz Tański, Weronika Smok, Oleg Polishchuk

**Affiliations:** 1 Institute of Engineering Materials and Biomaterials, Silesian University of Technology, Konarskiego 18A, 44-100 Gliwice, Poland; 2 Department of Machines and Apparatus, Electromechanical and Power Systems, Khmelnytskyi National University, Instytutska Street 11, Khmelnytskyi, Ukraine

**Keywords:** Nanowires, Optical materials and structures

## Abstract

The aim of the study was to prepare SnO_2_ nanowires via a combination of electrospinning and the sol–gel method from a polyvinylpyrrolidone (PVP)/dimetylformamide (DMF)/ethanol(EtOH)/tin(IV) chloride pentahydrate (SnCl_4_·5H_2_O) solution. The morphology, structure and chemical composition of the obtained PVP/SnO_2_ nanofibers and SnO_2_ nanowires were examined using transmission electron microscopy (TEM), Fourier transform infrared spectroscopy (FTIR) as well as a scanning electron microscope (SEM) with an energy dispersive spectrometer (EDX). The optical property analysis was performed on the basis of UV–Vis spectra of absorbance as a function of the wavelength, based on which the rated values of band gaps of the fabricated 1D nanostructures were determined. The morphology analysis showed that the obtained amorphous SnO_2_ nanowires with crystalline protuberances were characterized by a diameter of 50 to 120 nm. Results demonstrated that nanowires with a ratio of 1:1 precursor to polymer in the spinning solution were characterized by the smallest diameter after calcination and the smallest energy gap of 3.3 eV among all investigated samples. The rest of the studied materials were characterized by a larger energy gap (3.8 and 3.9 eV).

## Introduction

In recent years, the attention of scientists in the field of nanotechnology has been attracted by one-dimensional (1D) semiconductor nanomaterials based on metal oxides, which due to the large specific surface area, unique optical and electrical properties, can be applied in chemical sensors, batteries, fuel and solar cells, optic and optoelectronic devices or photocatalysis^1–8^. This group of materials includes SnO_2_, Bi_2_O_3_, TiO_2_, ZnO^9–12^. Among the many methods of their preparation such as the sol–gel method, chemical deposition, hydrothermal methods and magnetron sputtering, electrospinning method stands out^13–17^. The combination of the following two techniques of sol–gel and electrospinning used to prepare inorganic 1D nanostructures from metal oxides is gaining more and more interest due to the high quality of the product, simplicity, low manufacturing costs and a variety of available ceramic materials^18,19^.

In terms of optical properties, tin oxide is one of the most interesting materials. Tin oxide is a semiconductor with a wide energy gap of about 3.1–3.7 eV (Table 1), it is characterized by optical transparency as well as chemical and thermal stability^20,21^. This material is successfully produced in the form of thin layers, nanoparticles or porous microstructures, and is used mainly in gas detection, which owes structural stability, easy accessibility, low costs of manufacturing and high gas sensitivity^22–27^.

**Table 1:** Dependence of the energy band gap on the synthesis method and structure of SnO_2_.

Material type	Structure	Synthesis method	Band gap (eV)	References
Thin films	Amorphous	Atomic layer deposition (ALD)	3.75	^23^
Thin films	Tetragonal rutile	Spray pyrolysis	3.96–3.99	^28^
Nanopowder	Tetragonal rutile	Self-propagating high-temperature synthesis (SHS)	3.58–4.0	^29^
Nanoparticles	Tetragonal rutile	Sol–gel	3.9	^30^
Nanospheres	Tetragonal rutile	Hydrothermal	3.7	^31^
Nanorods			3.55	
Nanorods	Tetragonal rutile	Low temperature hydrothermal synthesis	3.88	^32^
Nanowires	Tetragonal rutile	Thermal evaporation	3.33	^33^
Nanofibers	Tetragonal rutile	Electrospinning	3.48	^34^
Nanofibers	Tetragonal rutile	Electrospinning	3.59	^35^
Nanofibers	Tetragonal rutile—amorphous	Electrospinning	3.3–3.9	*

*Presented work: “Synthesis of hybrid amorphous/crystalline SnO_2_ 1D nanostructures: investigation of morphology, structure and optical properties”.

The first reports on the production of SnO_2_ nanofibers by electrospinning from a solution were presented in 2006 by Dharmaraj with his colleagues. The electrospinning process of PVA, DMF, EtOH and SnCl_2_·2H_2_O solution was carried out using fixed parameters: a distance of 16 cm and a potential difference between the electrodes of 15 kV, resulting in composite PVP/precursor nanofibers. These nanofibers were calcined for 4 h at four different temperatures of 300, 400, 500 and 600 °C to assess the effect of the process temperature on the morphology and structure of the obtained materials. SEM and FTIR analysis showed that only the maximum temperature of 600 °C allowed the polymer to be removed to obtain fully crystalline fibers^36^. Two years later, i.e. in 2008, the work of a Chinese research team was published, in which the preparation of SnO_2_ nanofibers by electrospinning was presented and the applicability of this nanomaterial as a gas sensor was indicated. The nanofibers were obtained from a mixture of PVA, DMF, EtOH and SnCl_4_·5H_2_O using the following process parameters: distance and voltage between the electrodes of 0.5 cm and 5 kV, respectively, after which they were dried at 100 °C for 24 h. Then, the obtained PVA/SnCl_4_·5H_2_O nanofibers were annealed at three different temperatures of 300, 500 and 700 °C for 4 h. The authors, after analyzing the morphology of samples obtained from solutions with different PVP concentrations and different annealing temperatures, observed that fine, crystalline samples with a 6% share of PVP, free from defects and with a diameter of approx. 100 nm were obtained by calcination at 700 °C. The properties of the SnO_2_-coated ethanol detection sensor were tested, and it was shown that the newly developed sensor had a low detection limit, fast response and high repeatability^37^. In 2009, Qi et al. described a study of the impact of using a P123 block copolymer, as an addition to a spinning solution of PVP, DMF, EtOH and SnCl_2_·2H_2_O, on the morphology, structure and detection efficiency of NH_3_, C_2_H_5_OH, and CH_3_COCH_3_ by SnO_2_ nanofibers produced by electrospinning. On the basis of the observation of the morphology, structure and BET specific surface measurement, it was noticed that fibers with the addition of P123 have a diameter comparable to undoped ranging from 80 to 150 nm, but also have a much larger surface to volume ratio, hence much better sensory properties^38^.

Based on the authors' knowledge and experience, and a review of the literature, it has been noticed that one-dimensional nanomaterials characterized by both crystalline and amorphous structure may exhibit different optical, electrochemical properties as compared to those presented by completely crystalline or amorphous nanomaterials^5,12,39–45^. Obtaining such a structure depends largely on the calcination temperature. Zhou et al. showed that it is possible to control the crystallinity of Ge_2_O_3_ nanofibers using a different range of calcination temperatures—the higher it is, the higher the crystallinity of the nanomaterial^42^. As already mentioned, there is not much research carried out in this particular field so far, however, existing research indicates that such a combination of structures positively affects the energy band gap and the transition of electrons between energy states^12^. Therefore, we strive to pay special attention to these types of materials, because due to their unique properties they can be extremely valuable when used in modern optoelectronic devices.

Thus, the essence of the work is to produce hybrid crystalline-amorphous SnO_2_ nanowires and to determine the effect of the applied parameters of the electrospinning and calcining process on the morphology and structure of one-dimensional SnO_2_ nanostructures, with particular emphasis on the effect of the structure of this nanomaterial on its optical properties. Hence, in this paper the following studies are presented: characterization of the morphology, structure and chemical composition of the obtained nanomaterials using scanning and transmission electron microscopy (SEM and TEM), X-ray energy dispersion spectroscopy (EDX) and FTIR depending on the concentration of the tin oxide precursor and calcination temperature. In addition, the optical properties were analyzed on the basis of the UV–Vis spectrum and the energy gap for the SnO_2_ nanowires was calculated.

## Materials and methods

In order to prepare the spinning solutions, the following reagents were used: poly(vinylpyrrolidone) (PVP, 99%, Mw = 1,300,000 g·mol^−1^ provided by Sigma Aldrich) as a polymer matrix, ethanol (EtOH, 99.8% purity, purchased from Avantor Performance Materials Poland), *N*,*N*-dimethylformamide (DMF, 99.8% purity) and tin (IV) chloride pentahydrate (SnCl_4_·5H_2_O, purity of 98%) as an oxide precursor were purchased from Sigma-Aldrich.

The first step was to prepare four polyvinylpyrrolidone solutions by adding the polymer powder to ethanol in an amount corresponding to 8% wt. relative to the total weight of the solvents and stirring using a magnetic stirrer for 48 h. At the same time, various amounts of tin oxide precursor were dissolved in DMF and after 48 h of stirring was added to the PVP solutions and mixed for another 24 h to obtain homogeneous solutions with an EtOH:DMF mass ratio of 1:1 and PVP:SnCl_4_·5H_2_O mass ratio of 2:1, 1:1 and 1:3 (samples were designated as P1, P2 and P3, respectively).

**Figure 1:**
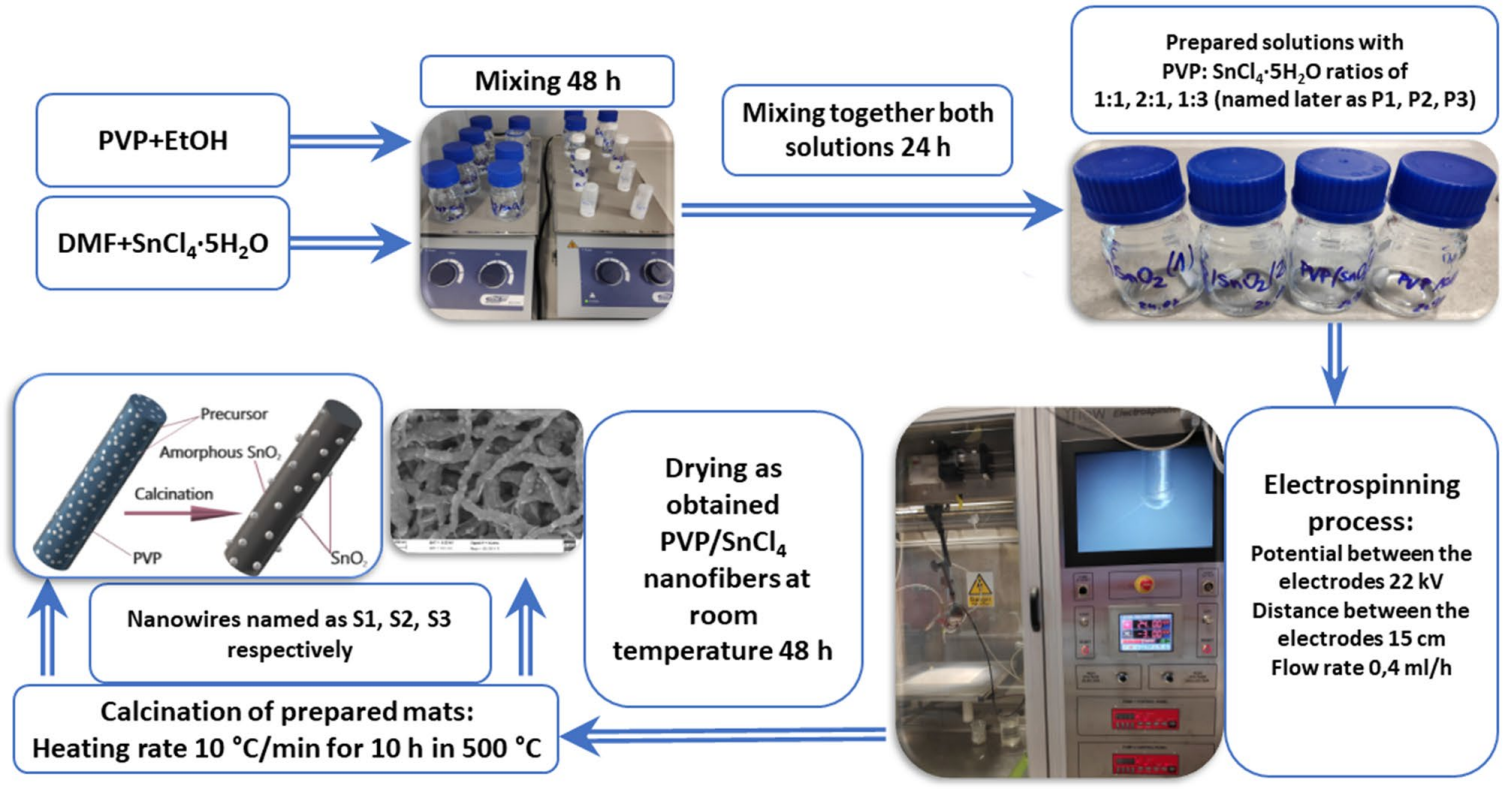
Preparation methodology scheme of PVP/SnCl_4_ nanofibers and SnO_2_ nanowires.

One-dimensional composite PVP/SnCl_4_ nanostructures were manufactured using FLOW—Nanotechnology Solutions Electrospinner 2.2.0-500 device, with the following parameters: voltage and distance between electrodes, a flow rate of 22 kV, 15 cm and 0.4 ml·h respectively. To degrade the polymer matrix, the obtained composite nanofibers were calcinated in a high-temperature vacuum furnace HT-2100-G-Vac-Graphit-Special, at a 500 °C temperature for 10 h, while the heating rate was 10 °C·min^−1^ for all samples; after that the samples were left in the furnace to cool. Nanowires corresponding to the ratios mentioned above were designated as S1, S2 and S3, respectively. A scheme summarizing the processes leading to the production of nanowires is shown in Fig. 1.

To investigate the morphology and structure of the produced materials, the (TEM) TITAN 80-300 FEI high-resolution transmission electron microscope was used for imaging in the transmission mode with the use of light and dark field (BF, DF), HAADF detector and filtration of energy, in particular using analytical electron microscopy in nanoareas.

Moreover, to study the effect of the heat treatment of composite PVP/SnCl_4_·5H_2_O nanofibers on the topography of the area and to analyze the chemical composition of the prepared samples, the Zeiss Supra 35 scanning electron microscope (SEM) with the EDAX Trident XM4 series X-ray spectrometer (EDX) were used. Based on SEM images, the diameters of the randomly selected composite and ceramic nanowires were measured using the Digital Micrograph. Fourier-Transform Infrared Spectroscopy (FTIR) spectra of the obtained nanomaterials were carried out by the spectrophotometer FTIR Nicolet 6700/8700.

Absorbance measurements have been conducted using the spectrophotometer UV–Vis Evolution 220 by Thermo-Scientific Company.ph software. To investigate the optical properties of the obtained composite and hybrid one-dimensional nanomaterials, the absorbance measurements, as a function of electromagnetic radiation falling on the sample were carried out using UV–Vis Evolution 220 spectrophotometer provided by Thermo-Scientific. Moreover, on the basis of UV–Vis absorption specters, the energy band gap value was determined.

## Results

### Analysis of morphology and structure

**Figure 2:**
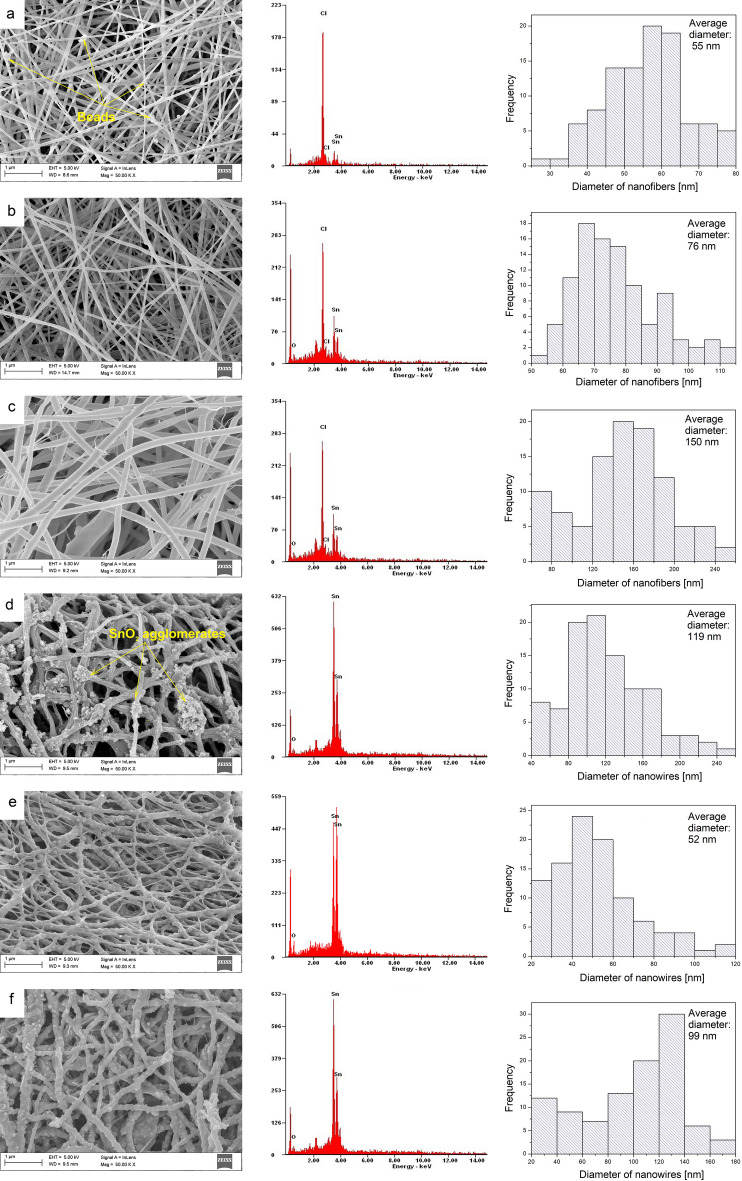
Left: SEM image of the topography on the surface of the formed fibrous composite mats and obtained bimodal nanowires, histograms presenting the distribution of 100-fold measurement of the diameter of randomly selected: nanofibers obtained from PVP:SnCl4 (**a**) 2:1, (**b**) 1:1, (**c**) 1:3 solutions and (**d**–**f**) corresponding them SnO_2_ nanowires after calcination in 500 °C, obtained EDX spectra from the entire area shown in the SEM images.

The topographic analysis of composite PVP/SnCl_4_ nanofibers marked as P2 and P3 revealed that both nanofibrous samples were free from defects in the form of beads, and their diameter was constant over the entire length of the fiber (Fig. 2b,c). Furthermore, the analysis of the P1 sample revealed a small number of beads, as well as glued fibers, which may be related to the hydrolysis and condensation reactions of the precursor at the mixing stage (Fig. 2a). This is a very common defect and at the same time undesirable, however it can be avoided at the preparation stage of the spinning solution by using anhydrous solutions or drying, as well as during electrospinning by controlling the process parameters appropriately^41^. It was found that all of the composite nanofibers have smooth surfaces and a cylindrical structure with an average diameter of 30–250 nm. This proves the proper viscosity of the spinning solution and the correctly established electrospinning process parameters.

A 100-fold measurement of the obtained PVP/SnCl_4_ random nanofibers confirmed that the diameter of the fibers electrospun from the solution with the lowest concentration of oxide precursor (P1) was the smallest compared to other samples and ranged from 30 to 80 nm, with nanofibers with diameters from 55 to 65 forming the largest group—39% (Fig. 2b). The increase in the precursor amount caused an increase in the diameter of the nanofibers, and in the case of sample P2, nanofibers with diameters in the range between 50–115 nm were observed, and the most common diameter values (also 39% of all measured diameters) were in the 65–85 nm range (Fig. 2c). The largest diameter was found in sample P3 obtained from a solution with six times more precursor than the second one. The nanofibers were characterized by diameters in the range of 60 to 250 nm, of which the most common values were in the 140–180 nm range (Fig. 2c). Therefore, it can be unequivocally stated that the precursor concentration is not without influence on the morphology of PVP/SnCl_4_ nanofibers.

S2 and S3 nanowires obtained after calcination at a 500 °C temperature, were characterized by a highly developed, rough surface with a large number of protuberances caused by crystallization on the surface, which is presented in Fig. 2e,f. Based on the significant similarity to the morphology of Ga_2_O_3_ nanofibers obtained by Zhou et al., S2 and S3 nanowires were found to be amorphous with SnO_2_ crystals on the surface, which is schematically verified and confirmed later by TEM analysis (Figs. 5, 6)^42^. The histograms shown in Fig. 2e,f reveal that in the case of both samples of S2 and S3 spinning solution, a decrease in the diameter of the nanowire was observed compared to the diameter of nanofibers before the heat treatment, which results from the degradation of PVP molecules during calcination^43^. The most numerous group (44%) of nanowires of the second sample were wires with a diameter value in the range of 40–60 nm, while for the third sample, as much as 50% of the measured diameters were in the range of 100–140 nm. In the case of sample S1, large agglomerates of SnO_2_ were visible on the wires, which results from defects in the form of beads already present before calcining. Moreover, nanowires were sintered together during calcination (Fig. 2d), so that, compared to nanofibers before calcination, a double increase in diameter was noticeable, of which the most common value—41%—was in the range of 80–120 nm (Fig. 2d; Table 2).

**Table 2:** The average diameter of PVP/SnCl_4_ nanofibers and SnO_2_ nanowires depending on the polymer to precursor ratio.

Type of nanomaterial	The average diameter of nanofibers (nm)		
	3 (1:3)	2 (1:1)	1 (2:1)
Before calcination PVP/SnCl_4_	150	55	76
After calcination SnO_2_	99	119	52

An analysis of the EDX spectrum of nanofibers obtained from each spinning solution showed the presence of SnCl_4_ precursor elements (Fig. 2a–c), while the spectra presented in Fig. 2d–f based on the samples after calcination confirmed obtaining pure SnO_2_ nanowires without the presence of undesirable elements (undescribed peaks near 2.0 eV presents Au and Pt, which were sputtered on the samples, Al comes from foil, and C from conductive carbon tape).

**Figure 3:**
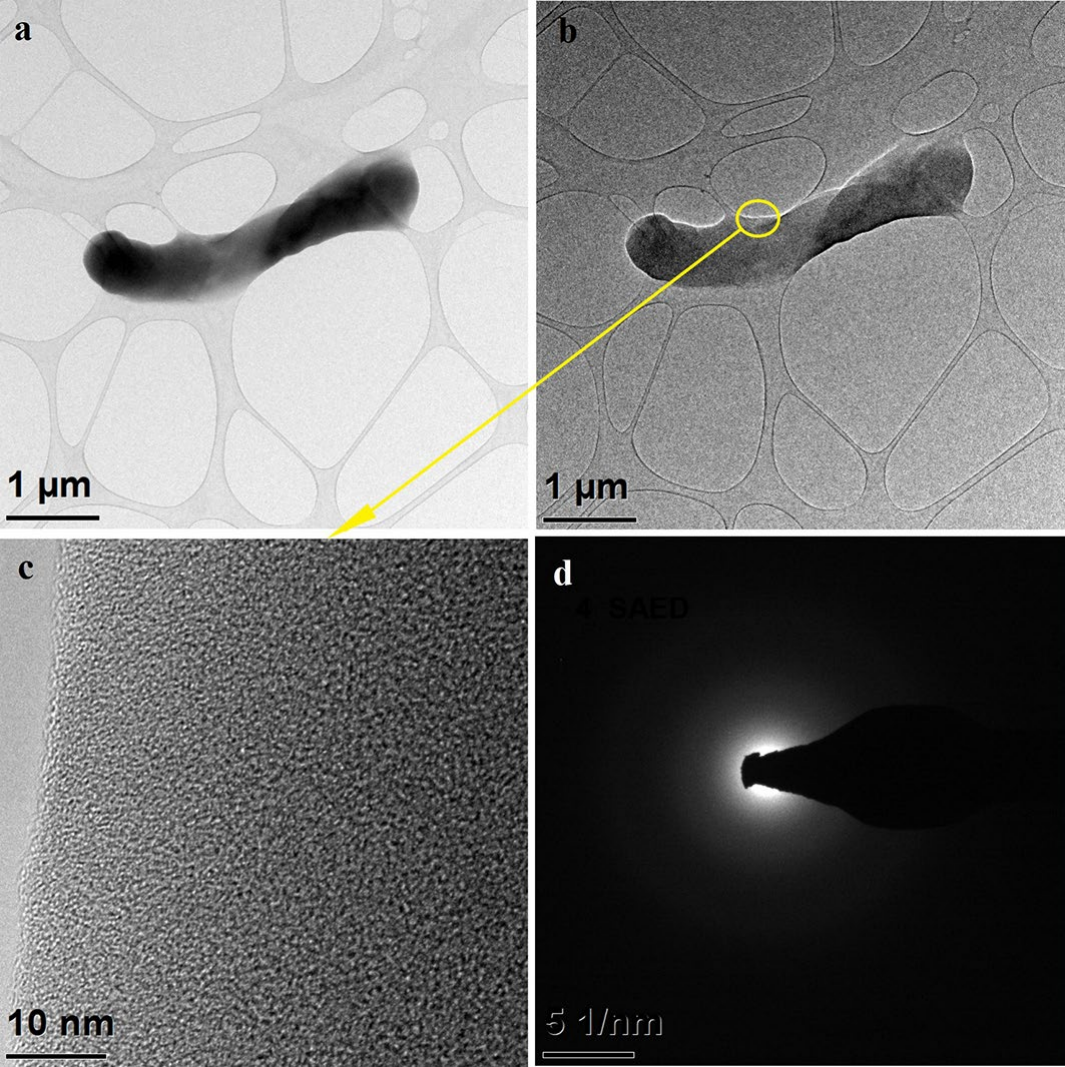
TEM images of the characterized 1D tin oxide nanostructures obtained by calcination P1 sample at 500 °C: (**a**) in bright field, (**b**) in dark field, (**c**) HRTEM of marked area and (**d**) diffraction image obtained using STEM mode in selected nanoareas.

**Figure 4:**
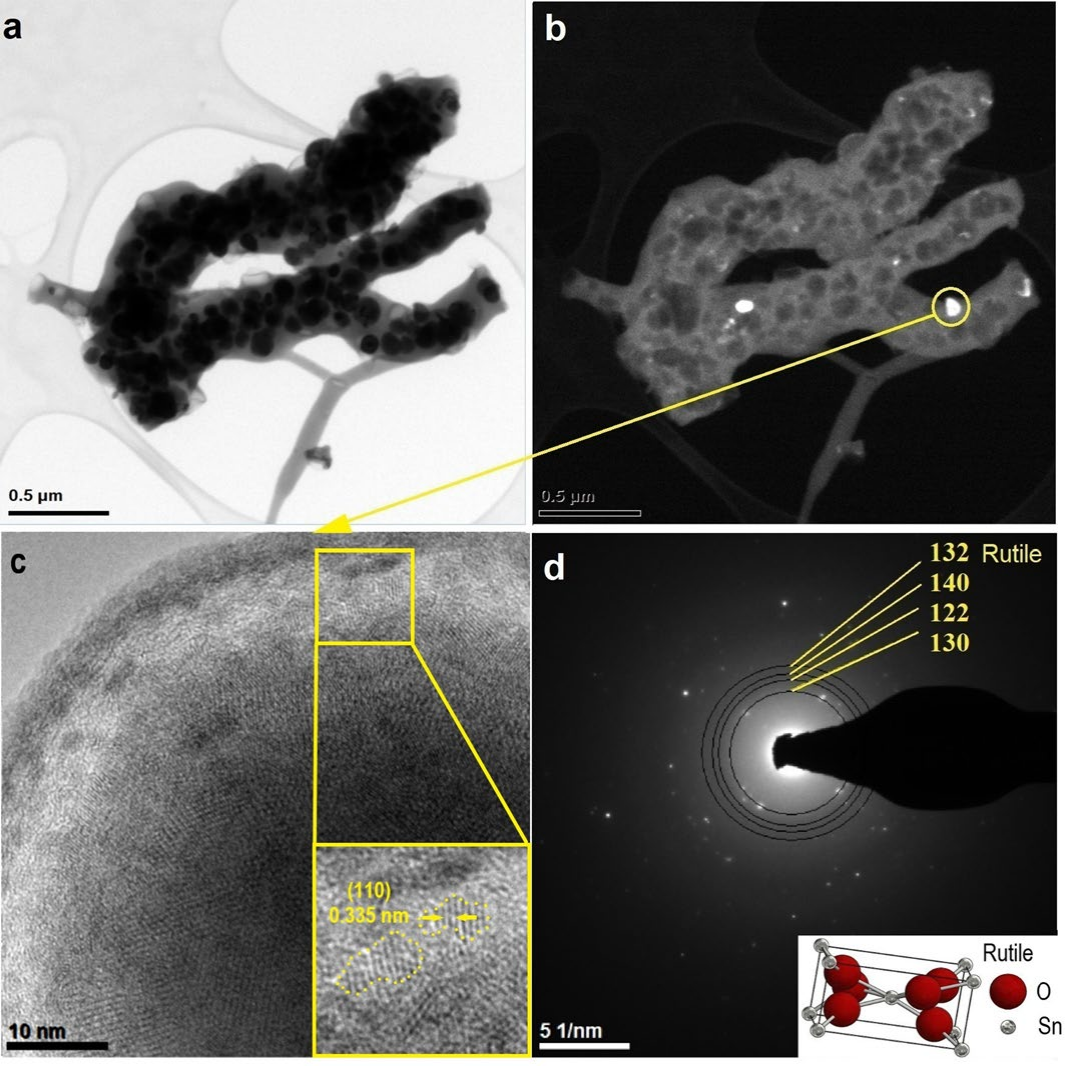
TEM images of the characterized 1D tin oxide nanostructures obtained by calcination P2 sample at 500 °C: (**a**) in bright field, (**b**) in dark field, (**c**) HRTEM of marked area with interplanar distance and (**d**) diffraction image obtained using STEM mode in selected nanoareas with resolved electronogram and rutile unit cell.

**Figure 5:**
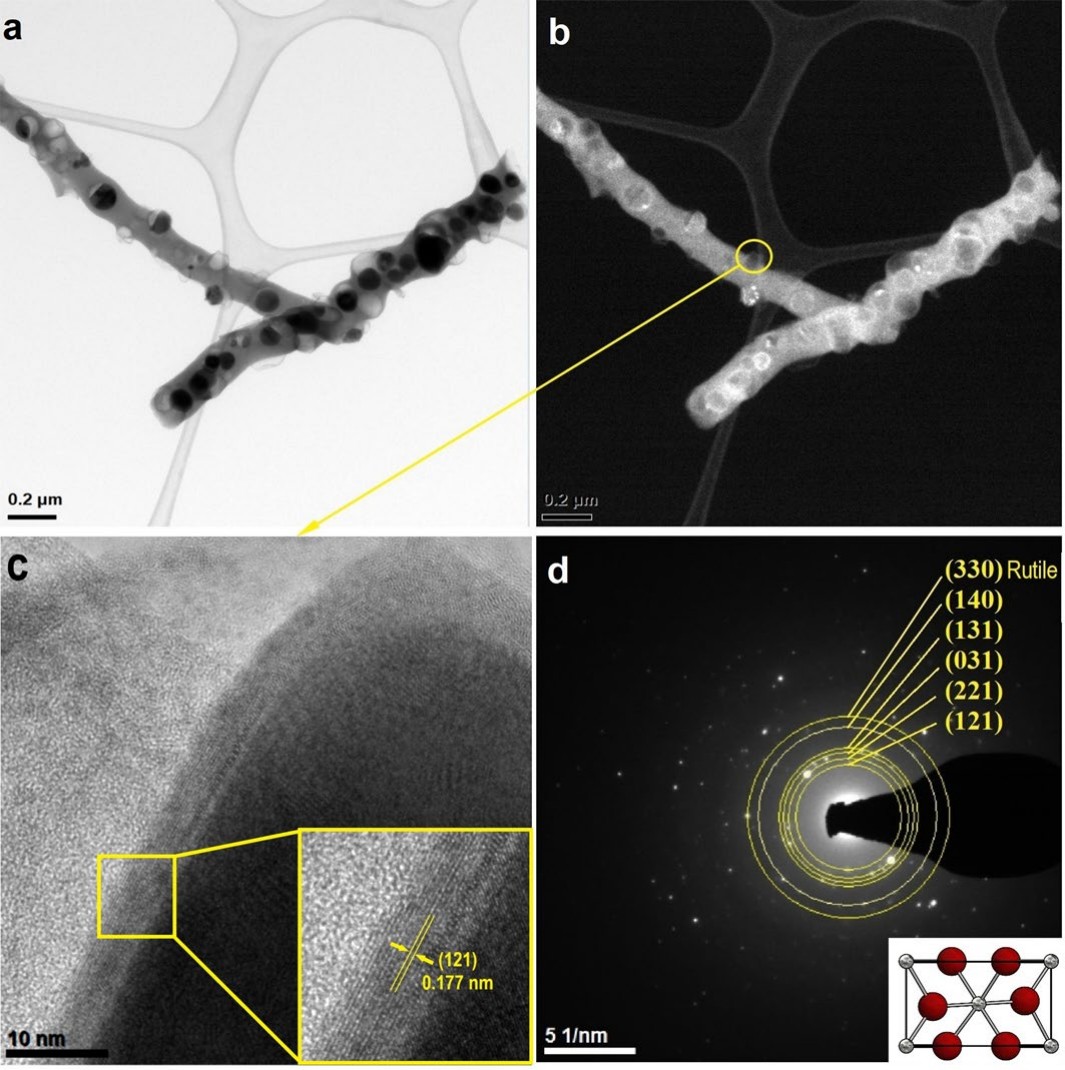
TEM images of the characterized 1D tin oxide nanostructures obtained by calcination P3 sample at 500 °C: (**a**) in bright field, (**b**) in dark field, (**c**) HRTEM of marked area with interplanar distance and (**d**) diffraction image obtained using STEM mode in selected nanoareas with resolved electronogram and unit cell presentation.

In order to investigate the morphology of the SnO_2_ nanowires obtained after calcination of PVP/SnCl_4_ nanofibers, the transmission electron microscope (TEM) characterization was carried out with the results presented in Figs. 3, 4, 5. Figure 3a,b reveal a smooth surface and the absence of any particles or roughness on a single S1 nanowire, which had a diameter of about 540 nm and a length of 3.5 mm. In the high-resolution mode, no distinct lattice planes fringes in the structure could be seen (Fig. 3c). Moreover, reflections on the diffraction spectrum obtained for a single SnO_2_ nanostructure were diffuse rings (Fig. 3d), which clearly indicates their homogenous amorphous structure.

Figure 4 presents TEM analysis for nanowires made from a spinning solution with the lowest precursor concentration, and in contrast to the S1 sample, branched and sintered nanowires with particles inside and on the surface of the nanowires were visible; the diameter of a single nanowire was determined to be 240 nm. In Fig. 4c, at high magnification of a single particle of the surface, it has been observed that an amorphous shell covers the polycrystalline particle and crystallographic lattice planes of different orientation were visible. The electronogram obtained from the area visible in Fig. 4c presents the diffraction image derived from a single particle on the surface of the nanowire and reflections in the form of diffraction rings deriving from the following planes with Miller indices: (130), (122), (140), and (132), which correspond to the tetragonal, rutile structure of SnO_2_ assigned to the spatial group P 42/m n m no. 136.

In the images taken in the bright and dark fields, two nanowires are visible, their diameter was about 130 nm and their length was about 2 µm, which makes it possible to state that the obtained material could be classified as one-dimensional (Fig. 5a,b). In Fig. 5a, as in the previous sample, dark crystalline protuberances are visible on the light amorphous wire. To further confirm the crystallinities of single surface protuberance, selected area electron diffraction (SAED) patterns were obtained from the area shown in Fig. 5c. Distinct ring patterns were indexed to the polycrystalline tetragonal structure of SnO_2_ from the following planes with Miller indices: (121), (221), (031), (131), (140) and (330), which were assigned to the spatial group P 42/m n m no. 136 (Fig. 5d).

It has been demonstrated that the nanowire protuberances seen in SEM images were polycrystalline rutile nanoparticles with diameters up to 90 nm, in which crystallization in the amorphous wire has been stopped^42^. The combination of SEM and TEM analysis allows to state that regardless of the precursor concentration, after calcination at a 500 °C temperature, amorphous SnO_2_ and amorphous-crystalline SnO_2_ nanowires were obtained (Figs. 2, 3, 4, 5).

**Figure 6:**
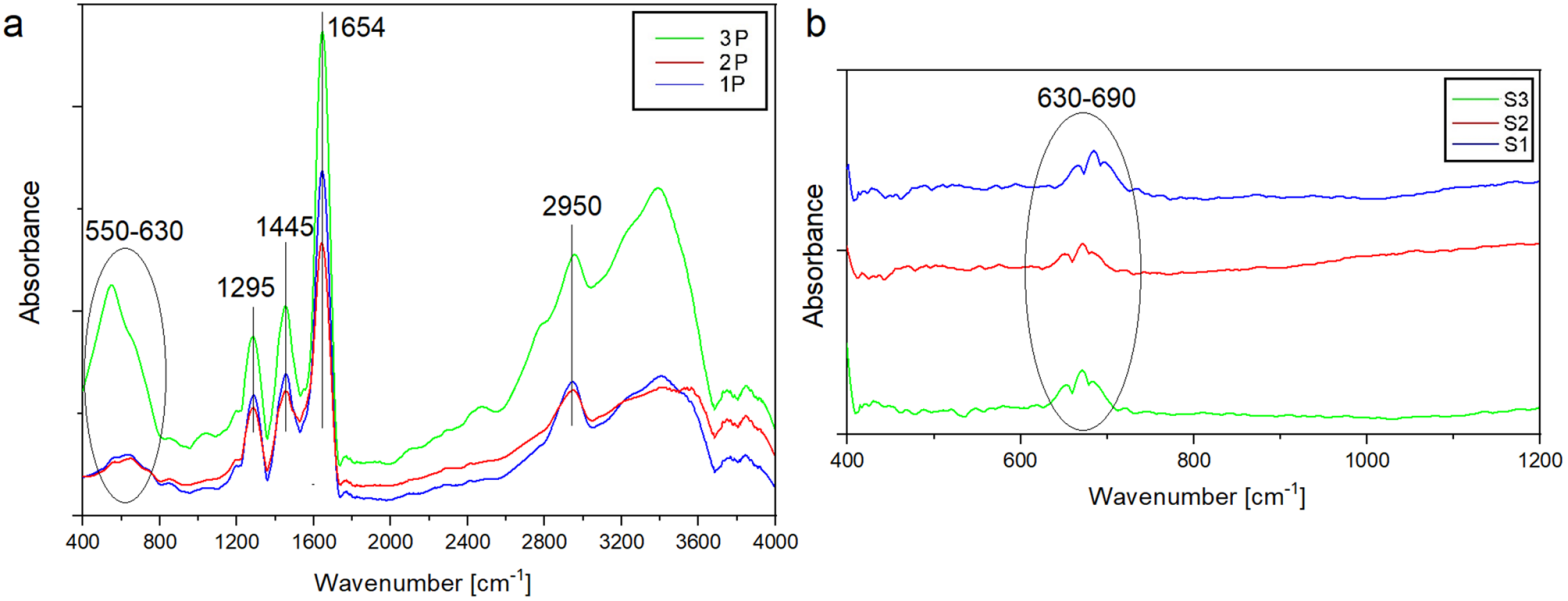
FT-IR spectra of as-prepared PVP/SnCl_4_ nanofibers and SnO_2_ nanowires calcined at 500 °C (the spectrum recorded for the nanowires is presented for the 400–1,200 cm^−1^ range due to background noise above this range).

In order to determine the structure of the obtained PVP/SnCl_4_ and SnO_2_ nanowires, an analysis using a Fourier-Transform Infrared spectrometer (FTIR) was performed (Fig. 6). In Fig. 6a, the characteristic absorption peaks of PVP were observed at 1,654, 1,445, 1,370 and 1,295 cm^−1^ which correspond to the vibration of the carbonyl group C=O, O–H bending and -C-N stretching, respectively (Fig. 6a). The strong peak observed at 2,950 cm^−1^ can be assigned to asymmetric stretching vibration of C–H bending. Moreover, the band at 550–60 cm^−1^ corresponds to the –Sn–O bond; higher absorbance in this range for sample P3 is associated with the highest concentration of precursor^46–48^. One visible wide peak in the spectrum recorded for SnO_2_ nanowires can be observed (Fig. 6b). The band appearing at 630–690 cm^−1^ is associated with the fundamental –Sn–O bond^36^.

### Analysis of optical properties

In order to investigate the optical properties of 1D SnO_2_ nanostructures obtained from solutions with three different precursor concentrations, UV–Vis spectral studies were performed. Based on the recorded absorbance spectra as a function of the electromagnetic radiation where the wavelength was in the range of 200–1,100 nm, the optical band gap of the manufactured nanowires was determined.

**Figure 7:**
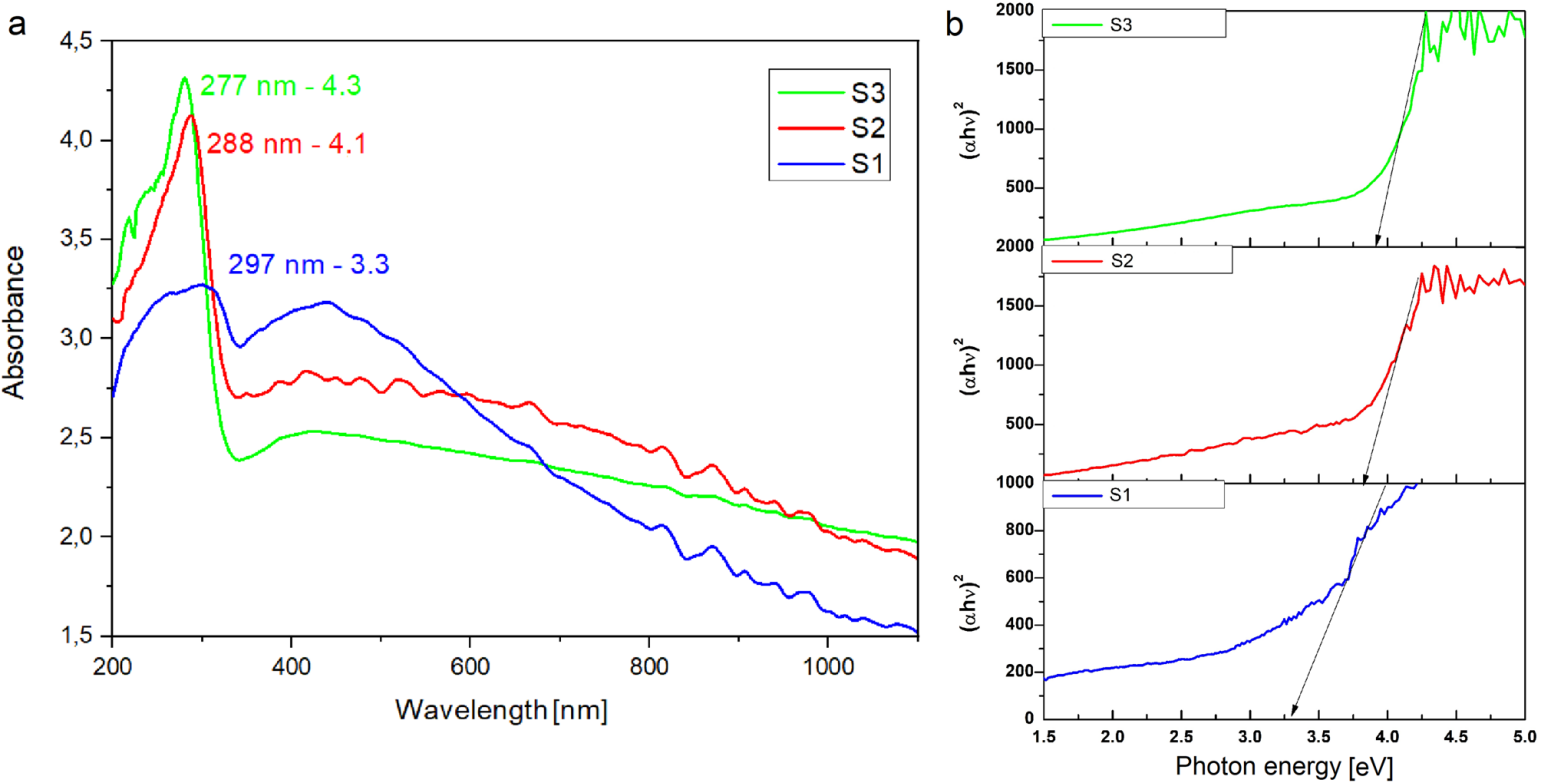
UV–Vis: (**a**) absorption spectrum of SnO_2_ nanowires calcined in 500 °C, (**b**) (αhν)^2^ versus photon energy (hν) plots, along with matching straights, crossing of which with energy axis corresponds to energy gap values of SnO_2_ nanowires.

Spectral characteristics recorded for tin oxide nanowires obtained from the second and third solutions showed the presence of a sharp absorption edge in the middle UV region, with the sharp absorption edge wavelength estimated at of 300 nm (Fig. 7a). The absorption maxima were registered with a wavelength of 288 and 277 nm, respectively. In Fig. 7a, it can be seen that the first sample shows a gradual fall in absorption in the UV region, wherein the absorption maxima could be identified at the characteristic wavelength of about 297 nm. The values of absorption edges obtained for SnO_2_ nanowires are similar to those obtained for SnO_2_ nanocrystals^36,49^. Moreover, the decrease in the maximum absorption for the first sample was recorded from 4.3 for the first sample to 3.3 in the middle UV range, which may be due to smaller values of nanowire diameters. Similarly, in the visible light range, a decrease in absorbance to 1.5 was noticed.

The results of studies on the interaction of SnO_2_ nanowires with electromagnetic radiation in the 200–1,100 nm range clearly indicate very effective absorption of ultraviolet radiation, which can be applied in photocatalytic reactions, manufacturing photovoltaic cells and layers protecting against radiation in this range.

The optical band gap of the obtained one-dimensional tin oxide nanostructures was determined with the help of the Tauc equation (1)^5^:

1 $$ \alpha {\text{h}}\upsilon \, = {\text{ A}}({\text{h}}\upsilon - {\text{E}}_{{\text{g}}} )^{\rho } $$

where, α—absorption coefficient, h—Planck constant, υ—electromagnetic radiation frequency, A—constant, hν—photon energy, Eg—energy band gap, ρ—coefficient, the taken value was ½.

Figure 7b presents the determination of the optical energy band gap based on the (αhν)^2^–(hν) plots. The optical band gaps of SnO_2_ nanowires obtained from the first, second and third solutions were 3.3, 3.8 and 3.9 eV, respectively. The nanowires marked as S2 and S3 were characterized by a much higher energy gap than the gap presented by S1, due to their smaller diameter size and shift of absorption edge. The hybrid SnO_2_ nanowires presented in this work have a significantly higher energy gap compared to the gap of other forms of SnO_2_, for example, in comparison to bulk SnO_2_ (3.6 eV) or thin films (3.54)^38,50^. The above results indicate a strong quantum confinement effect in SnO_2_ one dimensional nanomaterials. This is a typical phenomenon for semiconductor 1D nanostructure in which electrons are free to move in one direction, while quantization occurs in the remaining two directions and it is associated with change in the electronic state of the semiconductor. It leads to the widening of the forbidden band and the decreasing of density of electronic states at the edges of the conduction and valence band. This improves electron transfer between states through the position shift of the conduction band to more negative values^51^. Favorable optical properties makes them extremely desirable in construction of optoelectronic devices such as novel type dye sensitized solar cells (DSSC). The produced one-dimensional SnO_2_ nanomaterials can be an alternative to TiO_2_ as a DSSC photoanode material due to the large surface area and similar optical properties like energy gap and light absorption range^45^.

## Conclusions

In summary, hybrid amorphous-crystalline SnO_2_ one-dimensional nanomaterials have been successfully prepared by a combination of two techniques: sol–gel and electrospinning from PVP/DMF/EtOH/SnCl_4_·5H_2_O solution in order to determine the effect of precursor concentration on their structure, morphology and optical properties. Using the following polymer to precursor ratios: 1:1, 2:1, 1:3, composite PVP/SnCl_4_ nanofibers were produced in the electrospinning process and then calcined at the temperature of 500 °C for 10 h.

SEM analysis showed that after the heat treatment process, amorphous nanowires with crystalline SnO_2_ protuberances on the surface were obtained, but only for samples with higher precursor concentrations. Moreover, an increase in diameter from 55 to 150 nm of composite nanofibers with precursor concentration was observed. The nanowires obtained from the solution with the lowest precursor concentration were sintered and clusters of the ceramic phase were visible, which is the result of the presence of beads already in the composite fibrous mats. TEM analysis allowed to confirm that amorphous and amorphous nanowires with polycrystalline SnO_2_ nanoparticles were manufactured. The presence of Sn–O stretching in the IR spectra and no peaks from the precursor on the EDX spectrum on the samples after calcination at a 500 °C temperature confirmed the formation of tin oxide.

The analysis of the optical properties of the manufactured nanowires was performed based on the absorbance as a function of the wavelength specters using a UV–Vis spectrophotometer. In the spectrum, a high degree of absorption of electromagnetic radiation of the UV range was noticed in all samples. The band gaps of the calcined fabricated nanowires were calculated from optical spectra and ranged from 3.3 to 3.9 eV; the smallest value was obtained for a sample with a 2:1 PVP to precursor ratio, which is lower than other tin oxide nanostructures. Based on these results, it can be deducted that hybrid SnO_2_ nanowires with excellent optical properties could be used for the fabrication of novel types of solar cells, optoelectronic devices and as a photocatalyst in water treatment.

## References

[1] Yu L, Li C, Ma S, Li Y, Qi L, Yin M, Fan X (2019). Optoelectronic gas sensor sensitized by hierarchically structured ZnO nanorods/Ag nanofibers via on-chip fabrication. Mater. Lett..

[2] Tonezzer M, Kim JH, Lee JH, Iannotta S, Kim SS (2019). Predictive gas sensor based on thermal fingerprints from Pt-SnO_2_ nanowires. Sens. Actuators B Chem..

[3] Liu X, Jiang Y, Li K, Xu F, Zhang P, Ding Y (2019). Electrospun free-standing N-doped C@ SnO_2_ anode paper for flexible Li-ion batteries. Mater. Res. Bull..

[4] Haridas AK, Sharma CS, Hebalkar NY, Rao TN (2017). Nano-grained SnO_2_/Li_4_Ti_5_O_12_ composite hollow fibers via sol-gel/electrospinning as anode material for Li-ion batteries. Mater. Today Energy.

[5] Matysiak W, Tański T, Zaborowska M (2018). Manufacturing process, characterization and optical investigation of amorphous 1D zinc oxide nanostructures. Appl. Surf. Sci..

[6] Zheng F, Zhu Z (2017). Preparation of the Au@ TiO_2_ nanofibers by one-step electrospinning for the composite photoanode of dye-sensitized solar cells. Mater. Chem. Phys..

[7] Osali S, Esfahani H, Karami H (2018). Effect of Al doping on crystallography and electro-optical properties of ZnO semiconductor thin films prepared by electrospinning. Solid State Sci..

[8] Suphankij S, Mekprasart W, Pecharapa W (2013). Photocatalytic of N-doped TiO_2_ nanofibers prepared by electrospinning. Energy Procedia.

[9] Wang D (2019). J Constructing hierarchical SnO_2_ nanofiber/nanosheets for efficient formaldehyde detection. Sens. Actuator B Chem..

[10] Wang C (2009). Electrospinning preparation, characterization and photocatalytic properties of Bi_2_O_3_ nanofibers. J. Colloid Interface Sci..

[11] Someswararao MV, Dubey RS, Subbarao PSV, Singh S (2018). Electrospinning process parameters dependent investigation of TiO_2_ nanofibers. Results Phys..

[12] Matysiak W, Tański T (2019). Novel bimodal ZnO (amorphous)/ZnO NPs (crystalline) electrospun 1D nanostructure and their optical characteristic. Appl. Surf. Sci..

[13] Shaposhnik A (2014). Comparison of ammonia sensing characteristics of individual SnO_2_ nanowire and SnO_2_ sol–gel nanocomposite. Procedia Eng..

[14] Son NT, Noh JS, Park S (2016). Role of ZnO thin film in the vertically aligned growth of ZnO nanorods by chemical bath deposition. Appl. Surf. Sci..

[15] Zhang KX (2018). Synthesis, structural and optical properties of silver nanoparticles uniformly decorated ZnO nanowires. Chem. Phys. Lett..

[16] Obreja P, Cristea D, Dinescu A, Romaniţan C (2019). Influence of surface substrates on the properties of ZnO nanowires synthesized by hydrothermal method. Appl. Surf. Sci..

[17] Sirota B (2012). Bismuth oxide photocatalytic nanostructures produced by magnetron sputtering deposition. Thin Solid Films.

[18] Wang C, Shao C, Liu Y, Zhang L (2008). Photocatalytic properties BiOCl and Bi_2_O_3_ nanofibers prepared by electrospinning. Scr. Mater..

[19] Zhao M (2010). Synthesis and optical properties of Mg-doped ZnO nanofibers prepared by electrospinning. J. Alloy Compd..

[20] Al-Saadi TM, Hussein BH, Hasan AB, Shehab AA (2019). Study the structural and optical properties of Cr doped SnO_2_ nanoparticles synthesized by sol–gel method. Energy Procedia.

[21] Subramanyam K, Sreelekha N, Murali G, Reddy DA, Vijayalakshmi RP (2014). Structural, optical and magnetic properties of Cr doped SnO_2_ nanoparticles stabilized with polyethylene glycol. Phys. B.

[22] Li QL, Zhang XH, Lin T, Gao KH (2018). Electrical transport properties of polycrystalline SnO_2_ thin films. J Alloy Compd..

[23] Bang JH (2019). Effect of microwave irradiation on the electrical and optical properties of SnO_2_ thin films. Ceram. Int..

[24] Horti NC (2018). Photoluminescence properties of SnO_2_ nanoparticles: effect of solvents. Optik..

[25] Zhang J, Gao L (2004). Synthesis and characterization of nanocrystalline tin oxide by sol–gel method. J. Solid State Chem..

[26] Li H (2018). Porous SnO_2_ hollow microspheres as anodes for high-performance lithium ion battery. Mater. Lett..

[27] Zhao Y (2014). One-step synthesis of SnO_2_ hollow microspheres and its gas sensing properties. Mater. Lett..

[28] Choudhury SP (2016). Facile synthesis of SnO_2_ thin film by spray pyrolysis technique, investigation of the structural, optical, electrical properties. Mater. Today Proc..

[29] Kamarulzaman N (2019). Anomalies in wide band gap SnO_2_ nanostructures. J. Solid State Chem..

[30] Ahmed AS, Singla ML, Tabassum S, Naqvi AH, Azam A (2011). Band gap narrowing and fluorescence properties of nickel doped SnO_2_ nanoparticles. J. Lumin..

[31] Li S, Yu L, Man X, Zhong J, Liao X, Sun W (2017). The Synthesis and band gap changes induced by the doping with rare-earth ions in nano-SnO_2_. Mater. Sci. Semicond. Process..

[32] Inderan V (2015). Synthesis and characterisations of SnO_2_ nanorods via low temperature hydrothermal method. Superlatt. Microst..

[33] Kumar V (2009). Copper doped SnO_2_ nanowires as highly sensitive H_2_S gas sensor. Sens. Actuator B Chem..

[34] Bakr Z (2018). Synergistic combination of electronic and electrical properties of SnO_2_ and TiO_2_ in a single SnO_2_–TiO_2_ composite nanofiber for dye-sensitized solar cells. Electrochim. Acta.

[35] Wang K, Qian Z, Guo W (2019). Multi-heterojunction of SnO_2_/Bi_2_O_3_/BiOI nanofibers: Facile fabrication with enhanced visible-light photocatalytic performance. Mater. Res..

[36] Dharmaraj N, Kim CH, Kim KW, Kim HY, Suh EK (2006). Spectral studies of SnO_2_ nanofibres prepared by electrospinning method. Spectrochim. Acta A.

[37] Zhang Y, He X, Li J, Huang F (2008). Fabrication and ethanol-sensing properties of micro gas sensor based on electrospun SnO_2_ nanofibers. Sens. Actuator B Chem..

[38] Qi Q, Zhang T, Liu L, Zheng X, Lu G (2009). Improved NH_3_, C_2_H_5_OH, and CH_3_COCH_3_ sensing properties of SnO_2_ nanofibers by adding block copolymer P123. Sens. Actuator B Chem..

[39] Matysiak W, Tański T (2019). Analysis of the morphology, structure and optical properties of 1D SiO_2_ nanostructures obtained with sol-gel and electrospinning methods. Appl. Surf..

[40] Jarka P, Tański T, Matysiak W, Krzemiński Ł, Hajduk B, Bilewicz M (2017). Manufacturing and investigation of surface morphology and optical properties of composite thin films reinforced by TiO_2_, Bi_2_O_3_ and SiO_2_ nanoparticles. Appl. Surf..

[41] Tański T, Matysiak W (2018). Synthesis of the novel type of bimodal ceramic nanowires from polymer and composite fibrous mats. Nanomaterials.

[42] Zhou T (2016). Enhanced yellow luminescence of amorphous Ga_2_O_3_ nanofibers with tunable crystallinity. Ceram. Int..

[43] Ma LA, Wei ZH (2018). Field emission properties of crystalline-SnO2 (core)/amorphous-SnO2 (shell) nanowire arrays on carbon paper. Mater. Lett..

[44] Yu Y, Gu L, Dhanabalan A, Chen CH, Wang C (2009). Three-dimensional porous amorphous SnO2 thin films as anodes for Li-ion batteries. Electrochim. Acta.

[45] Li L (2010). Electrospun porous SnO_2_ nanotubes as high capacity anode materials for lithium ion batteries. Electrochem. Commun..

[46] Du JS (2007). Synthesis of poly (N-vinylpyrrolidone) nanofibers containing gold nanoparticles via electrospinning technique. Chem. Res. Chin. Univ..

[47] Deniz AE, Vural HA, Ortaç B, Uyar T (2011). Gold nanoparticle/polymer nanofibrous composites by laser ablation and electrospinning. Mater. Lett..

[48] Morales FL, Zayas T, Contreras OE, Salgado L (2013). Effect of Sn precursor on the synthesis of SnO_2_ and Sb-doped SnO_2_ particles via polymeric precursor method. Front. Mater. Sci..

[49] Gu F (2003). Synthesis and luminescence properties of SnO_2_ nanoparticles. Chem. Phys. Lett..

[50] Gorley PM (2005). SnO_2_ films: formation, electrical and optical properties. Mater. Sci. Eng B Adv..

[51] Kumar, D. S., Kumar, B. J., Mahesh, H. M. Quantum nanostructures (QDs): an overview. In *Synthesis of Inorganic Nanomaterials* 59–88 (Woodhead Publishing, 2018).

